# FLOTROP, a massive contribution to plant diversity data for open ecosystems in northern tropical Africa

**DOI:** 10.1038/s41597-019-0120-8

**Published:** 2019-07-08

**Authors:** Simon Taugourdeau, Philippe Daget, Cyrille Chatelain, Daniel Mathieu, Xavier Juanes, Johann Huguenin, Alexandre Ickowicz

**Affiliations:** 1 UMR SELMET Univ Montpellier, CIRAD, INRA, SupAgro, F-34000 Montpellier, France; 2 CIRAD UMR SELMET—PPZS, Dakar, Senegal; 3 CNRS, F-34000 Montpellier, France; 4 Conservatoire et Jardin botaniques (CJB), Geneva, Switzerland; 5 Tela Botanica, Montpellier, F-34000 France; 6 CIRAD UMR SELMET, F-34000 Montpellier, France

**Keywords:** Plant ecology, Biodiversity

## Abstract

The FLOTROP dataset contains numerous plant observations (around 340,000 occurrences) in northern tropical Africa (from the 5^th^ to 25^th^ parallel north) in open ecosystems (savannah and steppe). They were collected by multiple collectors between 1920 and 2012 and were managed by Philippe Daget. These observations are probably the most important and unique source of plant distribution over the Sahel area. The data are now available in the Global Biodiversity Information Facility, Tela Botanica website, and as maps in the African Plant Database. For the overall area involved, this dataset has increased by 40% the data available in the GBIF. For some countries between the 15^th^ and 21^st^ parallel north, the FLOTROP dataset has increased available occurrences 10-fold compared to the data existing in the GBIF.

## Abstract (summary)

**Table:** 

Design Type(s)	data integration objective • biodiversity assessment objective • data collection and processing objective
Measurement Type(s)	Plants
Technology Type(s)	digital curation
Factor Type(s)	species • geographic location • temporal_interval
Sample Characteristic(s)	Viridiplantae • North Africa

Machine-accessible metadata file describing the reported data (ISA-Tab format)

## Background & Summary

Tropical northern Africa (herein defined as between the 5th and 25th parallel north) is mostly occupied by open ecosystems, such as steppe and savannah^1^. The vegetation in these ecosystems is consumed by animals^2^, either wildlife or livestock, and is also used by the local communities for food, energy or medicinal purposes^3^. The open ecosystems in tropical northern Africa are of great importance for the economy, food security and human well-being.

Plant diversity within these ecosystems is driven by many factors, such as the climate, soil, fire and grazing^4,5^. Plant diversity in these regions is being greatly impacted by global change^6–8^. Historical data are needed to understand species and diversity dynamics. The database presented in this work is the collection of numerous datasets gathered over the years.

At the outset, the FLOTROP database was intended to store all the data recorded by IEMVT (French institute for tropical livestock production and veterinary medicine, now part of CIRAD) in the sixties. In 1993, CIRAD and CNRS set up a project to collect a maximum of botanical surveys within these regions^9–11^.

Two software packages were created by the team to manage the database. The first was created under DOS then a second was started under Windows using the APL DYALOG language. Data were gathered and scanned between 1993 and 2016. We extracted the data from the software version. We shared the species occurrences recorded in the database on the Tela Botanica website (http://www.tela-botanica.org/) and in the GBIF database^12^. The dataset is available following this link: 10.15468/oxunf1.

## Methods

### Original data collection in the FLOTROP software

The different data were collected by the FLOTROP team, mostly Philippe Daget. Botanical surveys were collected from different sources: direct contact with the authors of the survey, collection of data from the supplementary materials of Masters or PhD theses, technical reports on research or development projects, books, etc. A large share of the survey was only available on paper and most of the collection work was to convert those data to digital format.

All the observations are geotagged. For most of the survey, the coordinates were not recorded with a GPS. The coordinates were extrapolated using the location indicated in the surveys (i.e. name of the town, distance along the road from a known point, etc.) and a topographical map. The surveys without any geographical information were not included in FLOTROP. The survey protocols drawn up for the surveys were classed in 8 different categories:

1. Phytosociological unweighted surveys: These surveys contained the list of species presented at one station on one date.
2. Phytosociological weighted surveys: These surveys contained the list of species. For each species, the authors gave a Braun Blanquet score^13^.
3. Ecological surveys with species cover. These surveys contained all the species and their relative soil covers assessed using the Daget-Poissonet method^14^.
4. Ecological surveys with species contributions. These surveys contained all the species and their relative contributions to the plant cover assessed using the Daget-Poissonet method^14^.
5. Dominant species surveys. These surveys contained all the dominant species of the plant communities.
6. Ecological surveys with the number of individuals. In these surveys, the number of individuals per species was recorded.
7. Botanical occurrences. In this protocol, only one or a few species in the vegetation were recorded.
8. Surveys in experimental fields. These surveys were carried out in experimental fields.

During all this collection work, the taxonomic referential was harmonized to the Lebrun Referential^15^.

### Extraction, clean up and dissemination of the bases

In 2016, we extracted all the surveys from the FLOTROP software coded in APL DYALOG, and transformed the information into a more available format (.txt format, thus access format).

We found various errors in the database due to encoding problems during extractions: a mismatch between the protocol used and the way that abundance was coded, an inversion between the abundance and the number of species, and some surveys that were mostly only a list of species but were extracted as a survey with abundance. In this case, for half of the species, the number of species was inserted in the abundance columns.

We ran several tests to check for such errors. “Surveys containing such an error were flagged up”:

1. In the surveys with a Braun Blanquet score, the abundances could only be coded as 0, 1, 2, 3, 4 or 5. Otherwise, the surveys for this protocol that had abundance values different from these five were considered problematic and were not published.
2. In the surveys with species cover or a contribution estimation (protocols 3 and 4), the abundance estimation could not be over 100 (as 100% of the vegetation). The surveys that contained at least one species with an abundance over 100% were flagged up as problematic.
3. For all the surveys, if the species number and abundance were both over 100%, we assumed that it was probably a situation where the species number was placed in both the abundance and in the species number.
4. In the surveys with species cover or a contribution estimation (protocols 3 and 4), we flagged up the surveys where the sum of abundances was over 100%.

These different flags did not necessarily mean that the survey was problematic, but it was a good indicator. For the time being, we have not included any surveys that were flagged up and have only published surveys that did not have these errors.

We plan to review all the flagged up, and possibly corrected, surveys. This second database will be issued at a later date and the abundance estimators will also be included in this second release.

We chose to update the species referential with the African Plant Database (APD), which is widely accepted for African studies and recommended by the Association of the Taxonomic Study of the Flora of Tropical Africa (AETFAT). The comparison between the FLOTROP and APD names used a Fuzzy Lookup process (Ms Excel add-in). The analysis was carried out on one field containing full names (species with authors), with a threshold over 75%. As a comparison of 8,337 × 250,000 matrix names induced computer difficultly, an initial comparison was made with only North African names and then with tropical names. About 800 names were visually checked because the similarity was very low (between 075–0.95%), and 70 names concerning introduced species where added to APD. However, for 18 species out of the 8,337 species described in the FLOTROP list, we did not find any correspondence with the APD database. These names were probably transcription errors or confused species in the different Floras, for which finding the right name is quite impossible. In these cases, the species were entered into the database with the name selected in the FLOTROP database. The information of the name referential is put in the identificationRemarks.

For most of the surveys, the occurrences of species were also associated with an estimation of their abundance (either a percentage or Braun Banquet score, or individual counts). We only entered the occurrences. The different estimations of species abundances are available on request by email.

## Data Records

This dataset was included in The Global Biodiversity Information Facility (GBIF)^16^. The data were also shared on the e-Flora of Tela Botanica (www.tela-botanica.org). The base included 342,698 occurrences for plant species in the African region between the 5^th^ and 25^th^ parallel north. In order to compare these datasets with the existing data shared in the GBIF, we extracted from the GBIF website, on 28 July 2017 (www.GBIF.org)^12^, all the species occurrences within geographical coordinates between the 5^th^ and 25^th^ parallel north and between longitudes 20°W and 52°E. We did not download the occurrences that were obtained from a fossil sample. From all these occurrences, we only selected the species within African countries contained in the FLOTROP area (list of countries in Table 1). In all, 801,900 plant occurrences are already present (without fossil specimens). The FLOTROP database will increase the GBIF base by 43% for the area as a whole.

**Table 1:** Distribution between the different countries of plant occurrences in the FLOTROP and GBIF databases.

Country	FLOTROP	GBIF	% increase due to FLOTROP
Burkina Faso	60261	52935	114%
Benin	20235	281684	7%
Central African Republic (North)	2862	6288	46%
Cote d’Ivoire	12516	51795	24%
Cameroon (North)	29859	54075	55%
Cape Verde	5631	1	563100%
Djibouti	2527	89	2839%
Algeria (South)	2056	1058	194%
Egypt (South)	208	1733	12%
Eritrea	381	2043	19%
Ethiopia (North)	2348	89203	3%
Ghana	2649	63682	4%
Gambia	0	2217	0%
Guinea	1588	24468	6%
Guinea Bissau	320	6000	5%
Liberia	1565	27152	6%
Libya (South)	853	1089	78%
Western Sahara (South)	257	40	643%
Mali	24591	29318	84%
Mauritania (South)	15074	2445	617%
Niger	45732	9381	487%
Nigeria	4939	34089	14%
Sierra Leone	61	8663	1%
Senegal	56722	18372	309%
Somalia (North)	783	2500	31%
Sudan	11962	11794	101%
Chad	29837	2849	1047%
Togo	5289	16937	31%

The percentage due to the inclusion of FLOTROP in GBIF is calculated in the last column.

Several variables are presented for each occurrence:

Regarding the way data are downloaded on the GBIF site (Source data or GBIF archive), some numbers presented here may vary (especially for the taxonomic description). The number here corresponds to the source data file.

**Fig. 1:**
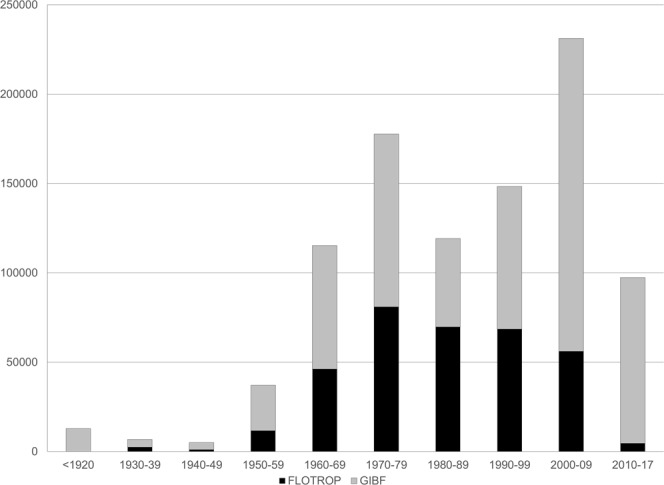
Temporal distribution of species occurrence in the FLOTROP database. Each bar represents the total number of occurrences now available in GBIF by decades. (In black the addition from FLOTROP and in grey the occurrences previously available in GBIF).

1. **Basis of record**: All the surveys were made based on human observation. On some occasions, herbarium samples were collected and were conserved in different herbariums, such as the ALF herbarium^17^ (http://publish.plantnet-project.org/project/herbieralf).
2. **The date of the observation (event Date)**: All the surveys are dated. However, for some surveys only the year was known. In this case, the date of the survey is 1st January of that year. Similarly, for some surveys the month and the year of the survey were known but not the day, so in this case the date of the survey is the first of the month. Figure 1 and Table 2 present the temporal distribution of the data per decade. The oldest observation in the FLOTROP database is from 1930. The data in FLOTROP mostly fall within the 1960–2000 period. The decade with the most data is the seventies, followed by the eighties. Severe drought events occurred during these two decades, especially in West Africa.

**Table 2:** Temporal distribution of FLOTROP and GBIF data by decades. The percentage increase in data is specified.

Period	FLOTROP	GBIF	% increase due to FLOTROP
<1920	0	12860	0%
1930–39	2427	4330	56%
1940–49	1010	4032	25%
1950–59	11609	25518	45%
1960–69	46173	69047	67%
1970–79	80952	96774	84%
1980–89	69739	49481	141%
1990–99	68549	79836	86%
2000–09	56041	175195	32%
2010–17	4606	92694	5%
3. **The country (country Code)**: The country is coded following the ISO 3166 guidelines. Regarding the distribution by country, in Table 1, we have presented the number of occurrences in the FLOTROP database by country and by the data currently available in the GBIF database. The contribution of FLOTROP is quite consequent for Sahel countries (increasing by 1000% the data available for Chad, 600% for Mali and Mauritania, 500% for Niger, 300% for Senegal and 100% for Burkina Faso and Sudan (including South Sudan)) and for two small countries that are not well documented, Cape Verde and Djibouti. For the southern area of the Saharan countries, the contribution of FLOTROP is also substantial, increasing the GBIF data by 12% for Egypt and 197% for Algeria. The contribution of FLOTROP is low for eastern Africa (an increase of only 3% of the GBIF data for Ethiopia) and for humid tropical western Africa. In tropical western Africa, the main ecosystem is tropical rainforest and FLOTROP only contains plant observations made in open ecosystems.
4. **The coordinates of the observation (decimal Latitude and decimal Longitude)**: All the surveys are geotagged with an accuracy to the minute (around 1.83 km in these regions). The coordinates are written in decimal for the latitude and the longitude. The climate within these African countries is highly variable from desert to tropical forest in Chad, for example. The North-South gradient in that country is a good indicator of the annual rainfall, especially in western Africa (dry climate in the North and wet climate in the South). Table 3 and Fig. 2 show the plant species observation number in FLOTROP and GBIF for each parallel. Between the 11^th^ and 25^th^ parallel, the inclusion of FLOTROP in GBIF will at least double the data (for the 11th parallel) or increase the data 19-fold (21st parallel). FLOTROP will thus amount to unique data on the drylands of sub-Saharan Africa. All the coordinates are in WGS 84. Figure 3 is a map of the density of occurrence of these species.

**Table 3:** Distribution between the different latitudes of plant occurrences in the FLOTROP and GBIF databases. The percentage due to the inclusion of FLOTROP in GBIF is calculated in the last column.

Latitude	FLOTROP	GBIF	% increase due to FLOTROP
5N	5466	75188	7%
6N	10683	134055	8%
7N	20247	100505	20%
8N	15229	52469	29%
9N	13532	79148	17%
10N	22888	68951	33%
11N	38964	39612	98%
12N	48311	21311	227%
13N	25865	17399	149%
14N	32843	9562	343%
15N	50144	4399	1140%
16N	31668	2766	1145%
17N	5804	952	610%
18N	4876	464	1051%
19N	5656	409	1383%
20N	2841	310	916%
21N	2257	122	1850%
22N	1490	411	363%
23N	1635	1447	113%
24N	707	450	157%

**Fig. 2:**
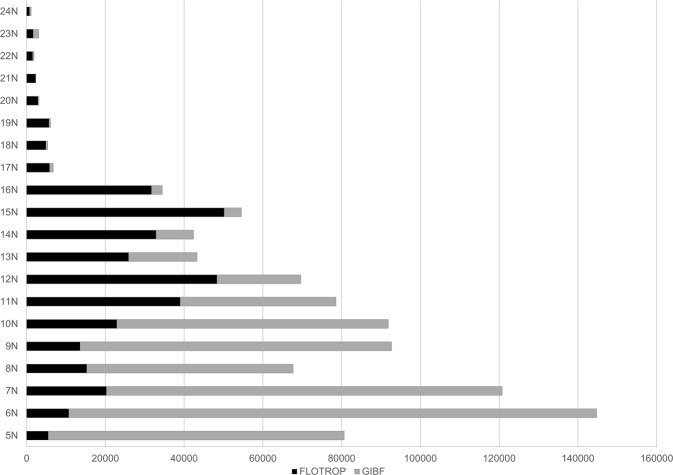
Latitudinal distribution of species occurrence in the FLOTROP database. Each bar represents the total number of occurrences now available in GBIF or the different parallels from the 5^th^ parallel N to the 25^th^ parallel N. (In black the addition from FLOTROP and in grey the occurrences previously available in GBIF).

**Fig. 3:**
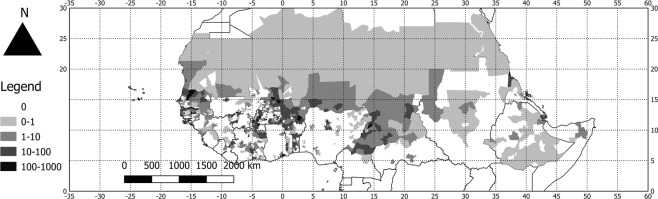
Map of the density of species occurrence in the FLOTROP dataset. number of occurrences per 100 km² for the different administrative units in the FLOTROP region.
5. **The name of species (scientific Name and taxon Rank)**: In all, 4,372 species are presented in the FLOTROP database, of which 64 species have more than 1,000 occurrences in the base. Figure 4 shows the number of occurrences for the 19 species that have more than 2,000 occurrences in the FLOTROP base and the number of occurrences that were previously in the GBIF base for the same region. Most of the species are at species level some only at genus level.

**Fig. 4:**
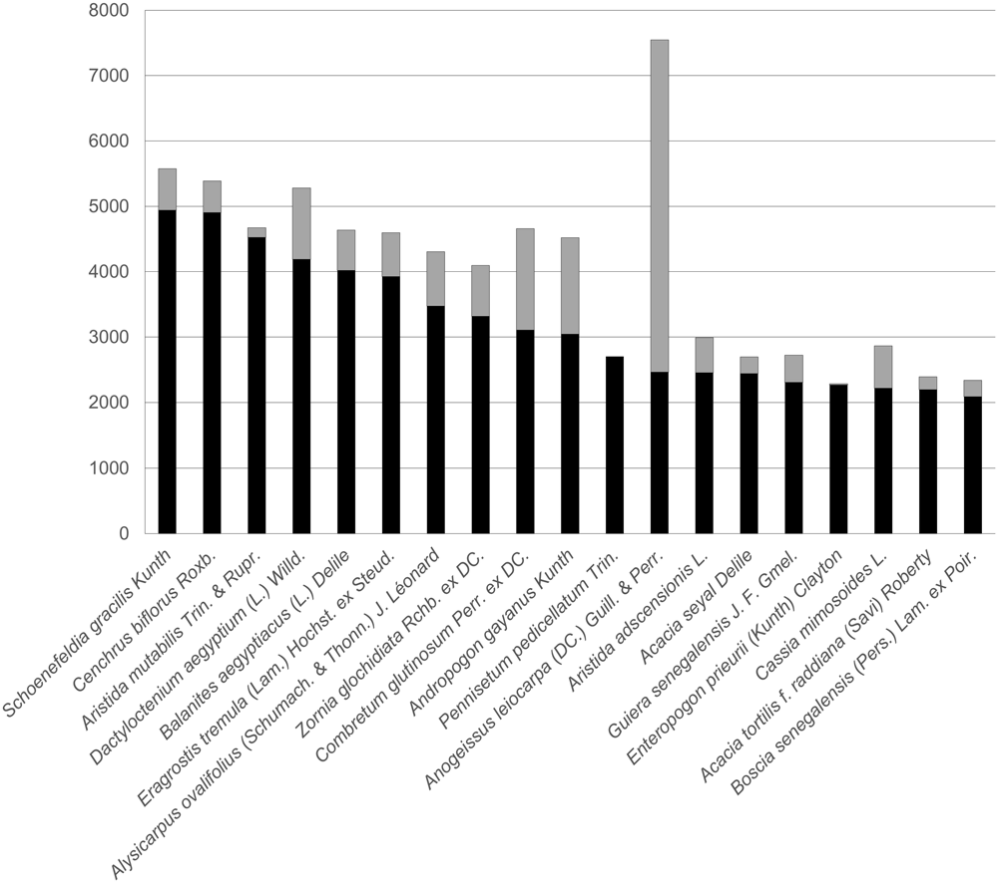
Number of occurrences for the most frequent species in FLOTROP. Each bar represents the total number of occurrences now available in GBIF for 19 species with more than 2,000 occurrences in the FLOTROP database (in black the addition from FLOTROP and in grey the occurrences previously available in GBIF).
6. **The name of the observer (recorded By)**: The authors of these different surveys are specified in the metadata. Only the last names of the authors are indicated in the database. In a few surveys, the author was not identified and was coded by an x. The FLOTROP database contains data obtained from 222 known different authors. The two authors who contributed most to the base are Jean Valenza and Bernard Toutain, with almost 30,000 observations in the base.
7. **Flotrop survey number (record Number)**: In the FLOTROP database, the observations have been regrouped per survey (observation of species together). The observations are in fact regrouped in 26,932 different surveys.
8. **Occurrence Remarks**: We add the original survey methods in remarks.
  Survey protocols are classed in 8 different categories. These protocols are coded from 1 to 8. Most of the data come from method 2, Phytosociological Weighted Surveys (262,270 occurrences). In all, 40,768 occurrences were obtained with Phytosociological Unweighted Surveys (method 1). The other method concerned fewer than 20,000 occurrences (see Methods for a full description of the methods).
9. **Identification Remarks**: This column contains the referential name used for the species names (either APD; original Flotrop name). Name in the APD with a doubt were coded with APD*.

## Technical Validation

Most of these field observations were connected by herbarium specimens validated by the IEMVT botanist, J.P. Lebrun, and now accessible in the CIRAD herbarium in Montpellier^17^ (about 50,000 specimens). Secondly, all the surveys included in the database were first assessed by Philippe Daget, who entered the surveys in the base according to their quality.

For the data obtained for FLOTROP, we carried out several tests to eliminate some encoding errors (see methods). We removed 90,000 occurrences for the upload, which will be corrected at a later date, based on expert knowledge.

The quantity of data is also a factor of quality: indeed, one species occurrence can be compared with the whole set of occurrences for the same species, to find out the distribution of any outliers.

Some of the original paper surveys included in the database are stored at CIRAD, in Montpellier. Where necessary, users can check the original if they need confirmation on the data.

## Usage Notes

The data presented here are a collection of multiple data sources, so they need to be analysed, using appropriate statistical tools.

Users should remember that plant identification and taxonomy in Mauritania, Senegal, Mali and Niger are based on old Floras. Despite the large number of observations of this dataset and the updated nomenclature used, many species are only known by one or two specimens, sometimes without all the organs that are useful for good identification. New collections and observations are absolutely needed to confirm the identity or ecology of some complex taxa.

The occurrence data can be used in at least two types of studies:

1. **Species distribution**: Despite the fact that these data are not a full inventory of each country, for the main reason that they focus on herbaceous vegetation used for livestock or on potential economic species, they can be used to update national inventories or checklists, as in new Flora projects for Chad or Burkina Faso.
2. **Species ecology**: These data can be used to understand the ecology of a species and its spatial and temporal dynamics. Many different models and statistical analysis can be carried out to study species^18,19^. For the spatial distribution of the species, users must be aware that the climate varied considerably in the West Africa over the period^7,8^ (1950–2010) and they must therefore include temporal changes in their spatial analysis model.
3. **Biodiversity studies**: These data can be used to assess some biodiversity indicators, such as alpha, beta and gamma diversity. Open ecosystems are composed of herbaceous and tree species. However, in the FLOTROP database some surveys were only carried out on herbaceous species, or on tree species. However, this information was not specified during the collection of the data. We therefore suggest that users take care when using the data where some surveys contain only one type of plant species. Moreover, due to the temporal differences and spatial life cycle of tree species and herbaceous species, it may be better to analyse both types of communities separately.

## ISA-Tab metadata file

Download metadata file.

## Data Availability

No specific codes were used to produce the data presented.

## References

[1] Le Houerou, H. N. The rangelands of the Sahel. *Journal of Range Management* 41–46 (1980).

[2] Assouma M (2018). How to better account for livestock diversity and fodder seasonality in assessing the fodder intake of livestock grazing semi-arid sub-Saharan Africa rangelands. Livestock Science.

[3] Fortin, D., Lô, M., Maynart, G. & Arseneault, C. *Plantes médicinales du Sahel*. (AGRIS, 1990).

[4] Hiernaux, P., de Leeuw, P. & Diarra, L. The interactive effects of rainfall, nutrient supply and defoliation on the herbage yields of Sahelian rangelands in north-east Mali. In *Proceedings of the International Conference on Livestock and Sustainable Nutrient Cycling in Mixed Farming Systems of Sub-Saharan Africa*. (Addis Ababa, Ethiopia, 1995).

[5] Hiernaux P (1998). Effects of grazing on plant species composition and spatial distribution in rangelands of the Sahel. Plant Ecology.

[6] Sinclair A, Fryxell J (1985). The Sahel of Africa: ecology of a disaster. Canadian Journal of Zoology.

[7] L’hote Y, Mahé G, Somé B, Triboulet JP (2002). Analysis of a Sahelian annual rainfall index from 1896 to 2000; the drought continues. Hydrological Sciences Journal.

[8] Nicholson, S. E. The West African Sahel: A review of recent studies on the rainfall regime and its interannual variability. *ISRN Meteorology* **2013** (2013).

[9] Tacher G (1993). Le projet FLOTROP au Cirad-emvt. Flotrop Info.

[10] Daget P, Gaston A (1999). Flotrop: constitution d’une base de données sur les pâturages d’Afrique tropicale septentrionale. Science et changements planétaires/Sécheresse.

[11] Daget P (1995). “FLOTROP”, une base de données agropastorales sur l’Afrique tropicale au CIRAD-EMVT. Journal of Tropical Livestock Science.

[12] GBIF.org. *GBIF Occurrence Download*, 10.15468/dl.utgo4l (2017).

[13] Braun-Blanquet, J. *Plant sociology: The study of plant communities*. 1st edn (McGraw-Hill Book Company, inc., 1932).

[14] Daget P, Poissonet J (1971). Une méthode d’analyse phytologique des prairies. Annales Agronomiques.

[15] Lebrun, J. *Enumération des plantes à fleurs d’Afrique tropicale*. (Conservation du jardin botanique de Genève, 1997).

[16] Taugourdeau S, Daget P (2018). The Global Biodiversity Information Facility.

[17] Daget P, Frohlich C (2001). L’herbier ALF en ligne. Systematics and Geography of Plants.

[18] Pearson RG (2007). Species’ distribution modeling for conservation educators and practitioners. Synthesis. American Museum of Natural History.

[19] Elith, J. & Leathwick, J. R. Species distribution models: ecological explanation and prediction across space and time. *Annual Review of Ecology*, *Evolution*, *and Systematics* **40** (2009).

